# Inclusion of tube current control in xy-plane for cardiovascular computed tomography reduces radiation exposure and produces better images

**DOI:** 10.1186/1749-8090-10-S1-A181

**Published:** 2015-12-16

**Authors:** Rong Hui (Misté) Chia, KH Toh, John WM Hoe

**Affiliations:** 1 Radiology Department, Mount Elizabeth Hospital, 228510, Singapore

## Background/Introduction

Radiation dose in cardiac scans may have an impact on incidence of cancer in patients. New techniques have evolved to include control in the xy-plane to produce more uniform images at lower doses.

## Aims/Objectives

This study investigates if the addition of tube current control in the xy-plane on image data (newer software) uses lower radiation dose and produces better cardiovascular computed tomography (CVCT) images, as compared to tube current control in the z-axis alone (traditional software).

## Method

Patients referred for CT angiography were randomly allocated to undergo scanning with a 320-row-detector CT using the traditional software and the newer software. Only those with no history of coronary revascularization; and no significant stenosis at the left main artery and proximal of left anterior descending artery were included for the study.

The magnitude of X-ray radiation exposure with the use of the traditional software was manually determined by radiographers according to protocol, while that for the use of the new software was automatically calculated to achieve a pre-set image quality. Estimated effective radiation dose was then calculated from the extended dose length product.

Two radiologists, blinded to the scanning parameters, assessed the overall image quality using a 5-point grading scale based on the quantity of mottle noise and streak artifacts.

## Results

From September 2009 to December 2011, 156 consecutive patients were recruited. Sixty-one patients underwent scanning using the traditional software while 95 patients had scanning with the newer software. Both groups were similar in baseline characteristics.

The use of the traditional software resulted in 55% more radiation exposure (mean radiation dose in mSv, traditional software: 3.1 ± 3.0; newer software: 2.0 ± 0.9). CVCT images produced with the use of the newer software were of better image quality (mean grade of image quality, traditional software: 2.6 ± 1.0; newer software: 3.8 ± 0.3).

## Discussion/Conclusion

Iterative reconstruction algorithms, involving the addition of tube current control in the xy-plane, use lower radiation dose and produce better quality CVCT images.

